# Blockchain technology in the smart city: a bibliometric review

**DOI:** 10.1007/s11135-021-01251-2

**Published:** 2021-10-06

**Authors:** Abderahman Rejeb, Karim Rejeb, Steven J. Simske, John G. Keogh

**Affiliations:** 1 Doctoral School of Regional Sciences and Business Administration, Széchenyi István University, Gyor, 9026 Hungary; 2 Higher Institute of Computer Science El Manar, 2, Rue Abou Raïhan El Bayrouni, 2080 Ariana, Tunisia; 3 Systems Engineering Department, Colorado State University, Fort Collins, CO USA; 4 Henley Business School, University of Reading, Greenlands, Henley-on-Thames, RG9 3AU UK

**Keywords:** Blockchain technology, Smart city, Bibliometric review, Co-citation analysis

## Abstract

Blockchain can function as a foundational technology with numerous applications in smart cities. The objective of this paper is twofold. First, it provides a detailed overview of the extant literature on blockchain applications in smart cities; second, it reveals the trends and suggests future research directions for scholars who wish to contribute to this rapidly growing field. We conducted a bibliometric review using a keyword co-occurrence network and article co-citation analysis. The analysis includes the assessment of 148 articles published between 2016 and 2020 in 76 academic journals. The review results demonstrate that the number of articles devoted to the study of blockchain applications and smart cities has increased exponentially in recent years. More importantly, the research identifies some of the most influential studies in this area. The paper discusses trends and highlights the challenges related to the deployment of blockchain in smart cities. To the authors’ best knowledge, this represents the first study to review the literature from leading journals on blockchain applications in smart cities using bibliometric techniques.

## Introduction

Recent advances in technologies have accelerated the development of smart cities. The smart city concept represents the next generation of urbanization (Li et al. 2016). Calvillo et al. (2016) described a smart city as "a *sustainable and efficient urban centre that provides a high quality of life to its inhabitants through optimal management of its resources.*" Aside from maintaining an advanced physical infrastructure, smart cities are characterized by the availability, integrity and quality of knowledge communication and social infrastructure (Caragliu et al. 2011).

In a smart city, advanced technologies and intelligent networks constitute the critical enablers for the effective functioning of the city. Critical components of a smart city ecosystem, including its broad infrastructure (e.g., mass transit, emergency services, energy grids) and government e-services (e.g., health services, permits, applications and approvals), interoperate in real-time. The wireless communication networks, coupled with self-organizing and self-healing networks, are critical in smart city realization. High-speed, real-time security protocols constitute a crucial component in the smart city ecosystem by providing the necessary security services in authentication, confidentiality, integrity and availability (Rathore et al. 2018).

Almost all smart applications continuously generate large amounts of data from heterogeneous sources. However, current database technologies do not effectively manage and securely store voluminous data. Sensors, devices, and vehicles connected through the Internet pose privacy risks and security concerns (Dwivedi et al. 2019). Although emerging technologies have overcome many infrastructure problems related to ageing infrastructure and the increasing demands of citizens for better services, the benefits of the smart city and urban improvement are not yet realized (Berglund et al. 2020). New technologies have paved the way for numerous opportunities for additional value creation; however, the progress of smart cities is still facing several challenges. A sustainable smart city should enhance the quality of life of its population through improved data security, privacy, efficient information sharing, effective decision-making, and high-quality services.

The emergence of blockchain technology promises vast improvements for smart city applications. According to Treiblmaier (2018, p. 547), blockchain is defined as "*a digital, decentralized and distributed ledger in which transactions are logged and added in chronological order with the goal of creating permanent and tamperproof records.*" Rejeb et al. (2018, 2019, 2020a, b) argue that blockchain constitutes a combination of multiple technologies, tools and methods that can be used for specific problems and business use cases. Blockchain technology has advanced as a potential solution to complex or persistent challenges within the smart city by enhancing transparency and providing a trust and accountability layer through its transaction immutability. Blockchain technology can transform smart city infrastructures, alter ecosystems for enhanced consumer services, and facilitate innovative applications. Blockchain is considered a new engine of growth and prosperity in the smart city as it enhances efficiencies, secures the communication of sensitive data, and increases the interoperability of smart city platforms. Besides underpinning cryptocurrencies, researchers and analysts envision that blockchain applications will span various sectors and help redefine global urban development through the protection of transactions and other services (Marsal-Llacuna 2018). Previous studies have attempted to review the existing applications of blockchain in the context of smart cities (Bernabe et al. 2019; Li et al. 2019), but to the best of our knowledge, no previous study has reviewed blockchain and smart city research using bibliometric tools such as keyword co-occurrence network and article co-citation analyses. This study provides a systematic bibliometric review on blockchain research from the smart city perspective to close this knowledge gap. In reviewing the literature, we aimed to understand the current state-of-the-art and fulfil the following objectives: reveal the evolution of blockchain and smart city research, identify the major contributing countries, and catalogue the publishing journals. The use of bibliometric tools serves to unfold the core content of blockchain-smart city research, the structure of co-cited articles, and the influential studies in the literature. Our main goal is to obtain a focused but detailed overview of the body of research. More specifically, in this investigation, we attempt to answer the following research questions (RQs):

- *RQ1* How did blockchain and smart city research evolve since their inception?
- *RQ2* What are the leading countries and journals publishing blockchain and smart city-related studies?
- *RQ3* What are the critical research hotspots in blockchain and smart city research?
- *RQ4* Which articles are the most influential in the academic literature?

This study is structured as five sections. After the introduction, we present the research methodology adopted in this study. Section 3 discusses the review's findings, including the temporal distribution of publications, the countries contributing to blockchain research, and the main publication outlets. In Sect. 4, we present the results of the keyword co-occurrence network along with the article co-citation analysis. Section 5 provides a discussion of current and future trends and challenges in blockchain-smart city research, followed by the limitations of the study, the potential research directions, and the managerial implications. The last section briefly concludes the paper.

## Research methodology

### Data collection

To investigate the current state-of-the-art of blockchain applications in the smart city, we conducted a bibliometric literature review. This type of review allows researchers to synthesize previous scholarly knowledge and inspire future research works. According to Álvarez-García et al. (2018), the power of bibliometric reviews lies in their capability to identify and classify a wide variety of documents within a specific area and to facilitate the analysis of information in order to show the trends based on synthesized data. Similarly, the use of bibliometric reviews ensures objectivity and offers unique insights into the literature (Koseoglu, 2016). Using bibliometric techniques, scholars can depict the conceptual space of a given research field and facilitate the interpretation of the findings.

The initial step of the bibliometric review consists of identifying the most suitable database for the study. For the selection of articles, we carried out searches in the Scopus database. The capability of the database to handle bibliographic references and quantify citations has made Scopus a widely used instrument for the analysis of any research field. Apart from this, Scopus is one of the most highly regarded academic databases globally, indexing approximately 70% more sources than the Web of Science (Brzezinski 2015). As per Harzing and Alakangas (2016), Scopus is one of the largest and trusted data repositories for peer-reviewed academic journals, books, chapters and conference proceedings covering various disciplines and depicting the dynamics of science and technology. We conducted our search using the title, abstract, and keywords fields by inserting the following query and a Boolean operator; Blockchain AND "smart cit*".

**Fig. 1:**
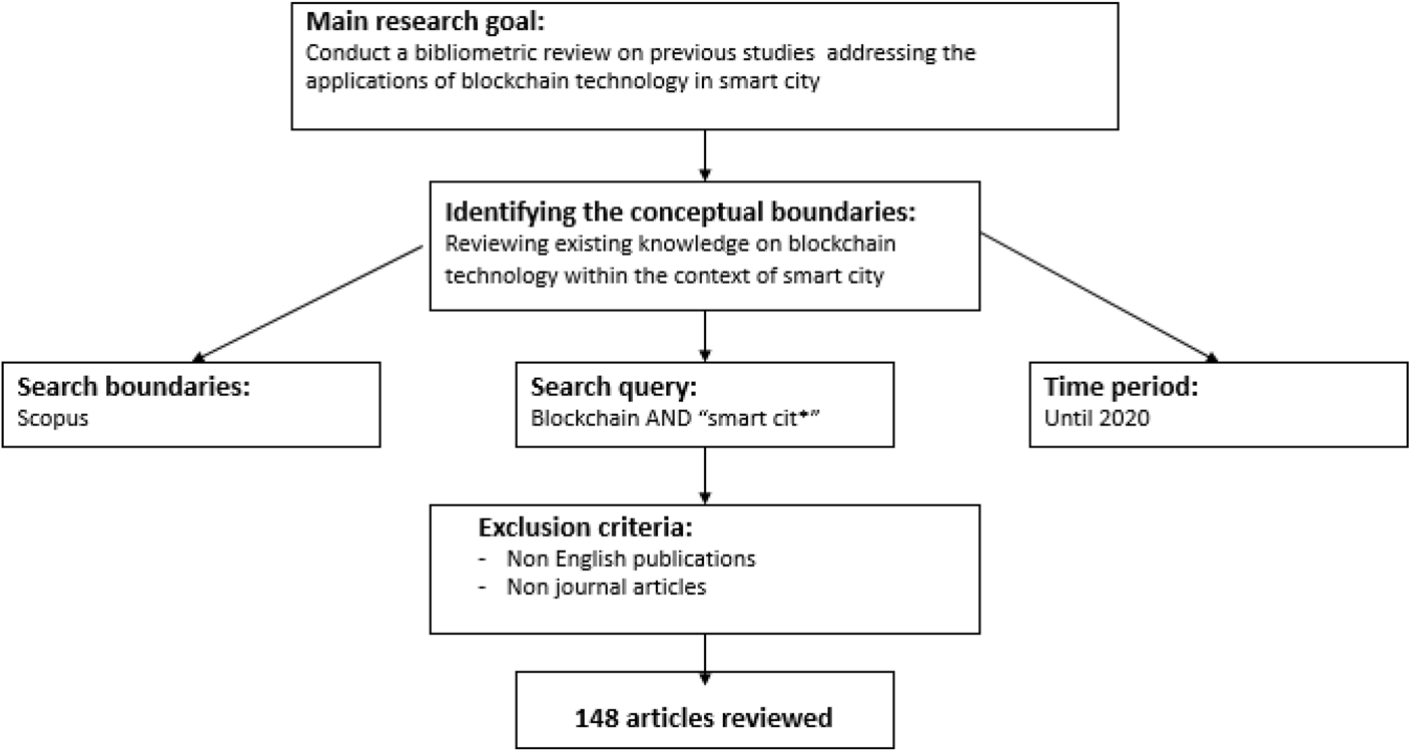
The literature review procedure and eligibility criteria

Using the outline of several studies as a reference, we considered only articles published in academic journals to analyze and develop bibliometric indicators. The selection of journal articles helps to ensure the reliability and academic nature of the analysis (Ramos-Rodríguez and Ruíz-Navarro 2004) because these resources provide a representative sample of international scientific activity. To widen coverage, we considered all publications in the English language. All journal articles published before 2021 were considered. After this step, the Scopus database returned 162 documents. To further refine the results, we limited the subject areas to engineering, computer science, social sciences, business, management and accounting, decision sciences, and economics. For clarity and transparency, the advanced search function used in Scopus is illustrated in Annex 1. Our review of past studies focused on identifying the connection of blockchain technology with the smart city. Therefore, priorities were given to the previously mentioned disciplines. The number of articles considered was reduced to 150. Each of these articles was carefully screened for relevance by reading the title, the abstract and the keywords. Eventually, 148 journal articles were retrieved for the final review and analysis (two of the 150 articles were considered “false positives” for the search result). Our sample included all articles that present the applications of blockchain technology in smart cities. The review procedure followed to obtain these articles, along with the eligibility criteria, is depicted in Fig. 1.

### Bibliometric methods

After the descriptive analysis of the selected publications, we carried out a deeper examination of the content (i.e., keywords) and the relationships among the articles to obtain more insights. Network analysis was employed based on bibliometric data to depict the network structure of blockchain-smart city research by using the visualization software VOSviewer (Eck and Waltman 2009).

A keyword co-occurrence analysis was performed to generate a more in-depth examination of the blockchain-smart city research field. This analysis helps to reveal the interaction between the different research directions and partition the field of knowledge (Ding et al. 2001). Unlike co-citation analysis, which is based on clustering the related references into groups according to their link strength (Zheng et al. 2017), keyword co-occurrence network analysis extracts author-supplied keywords in each publication and analyses their co-occurrence frequency (Ding et al. 2001). The high proximity of two keywords means that they are found in the same publications more frequently. To depict the structure of the field, VOSviewer was used because of its compatibility with Bibexcel and its ability to create the co-occurrence network. Moreover, we used the LinLog/modularity normalization method to display the clusters of keywords. This algorithm minimizes the distance between connected nodes, i.e., keywords (Newman 2004; Noack 2007).

Co-citation analysis was introduced by Small (1973). It was employed to investigate and visualize the dynamic aspects of blockchain-smart city research. The analysis of co-cited articles enables researchers to explore the links between such co-cited sources (Chen et al. 2010). As one of the most common relational techniques, article co-citation analysis assumes an association between two publications if they are both cited in subsequent studies (Small 1973). Co-citation patterns and frequency help identify knowledge domains since higher co-citation frequencies between two publications suggest stronger relationships, uniformity of references and collective knowledge (Fang et al. 2018). In this paper, VOSviewer was used to generate the article co-citation network. Initially, the output file from Bibexcel was loaded into VOSviewer. Several steps were followed to generate the article co-citation map. Before importing data to VOSviewer, we pre-processed the bibliometric data of our sample, and we set a threshold of two co-citations to be shown for the analysis. As a result, a document with a co-citation frequency below two is not considered in the co-citation analysis. As blockchain-smart city research is still in a nascent stage, we noted that setting a high threshold would lead to over filtering and the display of few articles in the visualization network. The clustering of research topics was displayed using normalization with the LinLog/modularity algorithm (Newman 2004; Noack 2007). The thickness of the edges reflects the strength of the relationship between co-cited articles.

## Findings

### Distribution of publications by year

**Fig. 2:**
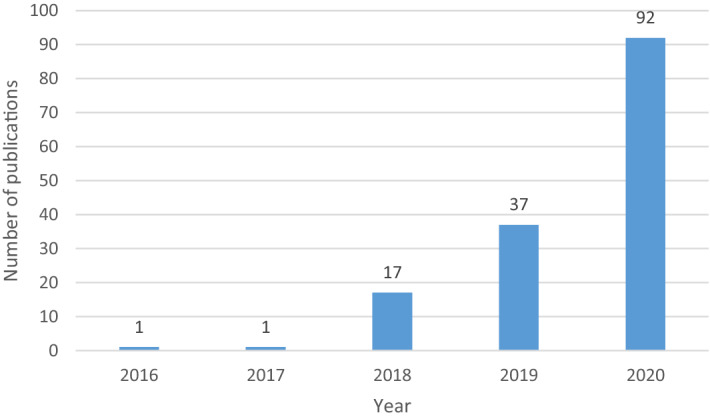
Year-wise distribution of publications

The search was performed on the 5th of January, 2021. Figure 2 illustrates the number of journal articles published by year and extracted from the execution of the research procedure. The first article that addresses the applications of blockchain technology in the smart city appeared in 2016. According to Rosati and Conti (2016), “smart city” has been used as a term since at least 2009 and several sources highlight that blockchain was first introduced in 2008. Our research reveals it took 7–8 years for the joint usage of blockchain and smart city in a research paper authored by Sun et al. (2016), who studied the contribution of blockchain technology in supporting the development of shared services in smart cities. In 2017, one article was published by Sharma et al. (2017), where the authors investigated the potentials of blockchain technology for intelligent transport systems. According to this study, blockchain could be used to design more intelligent, secure, distributed, and autonomous transport systems. From 2018 onward, there is a significant increase in the annual volume of articles, peaking in 2020 with 92 papers. As shown in Fig. 2, the number of articles published in 2018 increased significantly compared to the previous years. However, the scholarly output in 2019 and 2020 doubled from the previous year. Two factors can explain the growing interest level in blockchain applications in the smart city: first, the number of scholars worldwide has increased tremendously, concomitantly increasing the number of submissions to journals. Second, a high number of smart city-focused and blockchain-based decentralized applications are being developed (Kundu 2019). This finding is consistent with Li et al. (2019), who argue that smart energy and smart government applications are at an advanced stage with blockchain implementations. The interest in blockchain has also rapidly increased in other sectors such as healthcare (Bernabe et al. 2019), sharing services (Sun et al. 2016), transportation (Astarita et al. 2020), and energy (Makhdoom et al. 2020; Park et al. 2018; Tanwar et al. 2020). At the time of writing, we anticipate the number of publications incorporating blockchain technologies and smart cities will experience an exponential increase in the coming years, entering a ‘maturing phase’ of adoption and continued strong levels of academic attention and focus.

### Distribution of publications by country

**Fig. 3:**
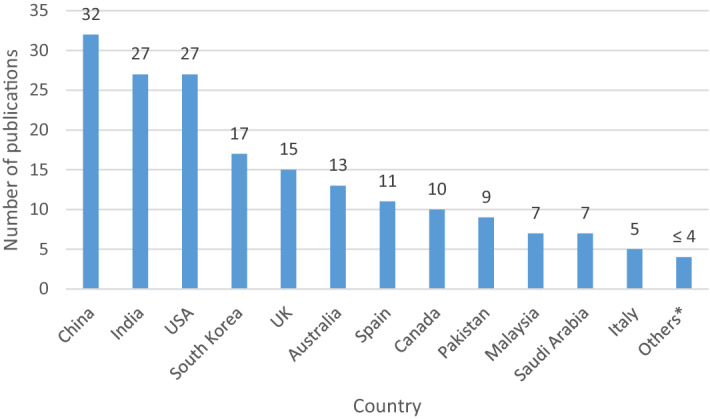
Country-wise distribution of publications

To study the country-wise distribution of the selected papers, we extracted the authors’ affiliations. From Fig. 3, it is observed that a significant contribution to the blockchain and smart city literature came from China, India and the USA, with 32, 27 and 27 papers each, respectively. This is not surprising as China has up to 300 cities with pilot projects for smart city construction (Sun et al. 2016). In such projects, blockchain is expected to enable smart cities and provide citizens with high-quality services. Related to the development of practical solutions for urban areas, China represents one of the leading countries that has promoted the adoption of blockchain for the authentication of data (Li et al. 2019), the security of imaging sensors for surveillance (Khan et al. 2020), the support of foreign exchange trade systems (Aggarwal et al. 2019), and the traceability of supply chains (Rejeb et al. 2020a, b, 2021a; Shen and Pena-Mora 2018). In India, Prime Minister Modi’s flagship project— the Smart Cities Mission— has placed a core emphasis on cutting-edge technologies such as blockchain in the country (Team Inc42 2018). The government of India has officially initiated the implementation of blockchain in the port city of Vishakhapatnam to secure digital transactions, maintain land records, and streamline automobile registrations (Bragadeesh and Umamakeswari 2018). The interest in blockchain has expanded rapidly in the past decade in the United States, where the scope of the technology extends from cryptocurrencies to public services, including accounting and taxation (Keibler 2019). The impetus for improved data management and space communications in various interplanetary space missions has urged the National Aeronautics and Space Administration (NASA) to consider blockchain technology for the development of an autonomous spacecraft system called the Resilient Networking and Computing Paradigm (RNCP) (Ismail and Materwala 2019).

Similarly, the Cook County Recorder of Deeds in Chicago initiated a project to test how digital property abstracts could be created and managed using blockchain (Kundu 2019). With the blockchain implementation, US State authorities and local governments aspire to improve the public and private sector services, enhance the relationship between government and citizens, and make operational and commissioning activities over smart urban infrastructures intrinsically secure and efficient. Scholars from South Korea have contributed to the literature with 17 studies. Researchers from the United Kingdom (UK), Australia, and Spain have also contributed substantially to the literature with 15, 13, and 11 papers, respectively. It should be noted that the category "Others" comprises all countries with less than five papers (total papers from such countries = 35).

### Distribution of publications by journal

**Fig. 4:**
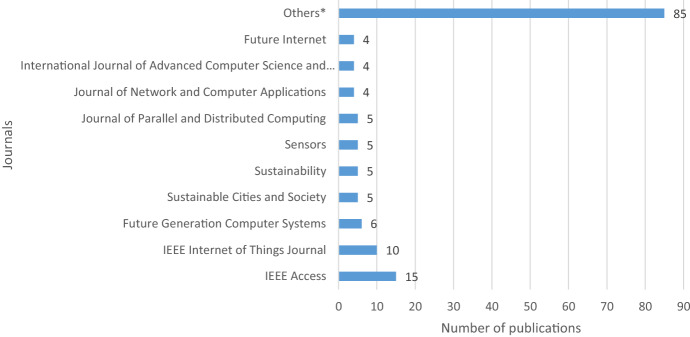
Distribution of publications by professional journals

The retrieved papers appeared in a wide variety of academic journals. Figure 4 presents the journals publishing on blockchain and the smart city and the respective number of articles. The 148 articles were published in 76 journals. In terms of output*, IEEE Access* was the journal publishing the highest number of articles (n = 15). *IEEE Internet of Things Journal* and *Future Generation Computer Systems* published 10 and 6 articles, respectively. The remaining journals on the list published either four or five articles each. The category "Others" consists of 66 journals that published less than four articles on blockchain applications in the smart city. In total, these journals published 85 papers. Taking a closer look at the scope of all journals, we observed that research on blockchain in the smart city context has received remarkable attention from the engineering and computer science communities and appeared in journals with a high impact factor.

Meanwhile, journals with a strong orientation toward management, business, urban sciences, and social sciences have published few articles on the subject. As a result, this suggests that the low output from these journals may be explained by either few submissions or high rejections if not aligned with the charter of the journal. This finding increases the utility of this study and makes this review invaluable for awakening the interest of social science scholars to examine the intricacies of blockchain research from the smart city perspective.

## Bibliometric analysis

### Keyword co-occurrence network

**Fig. 5:**
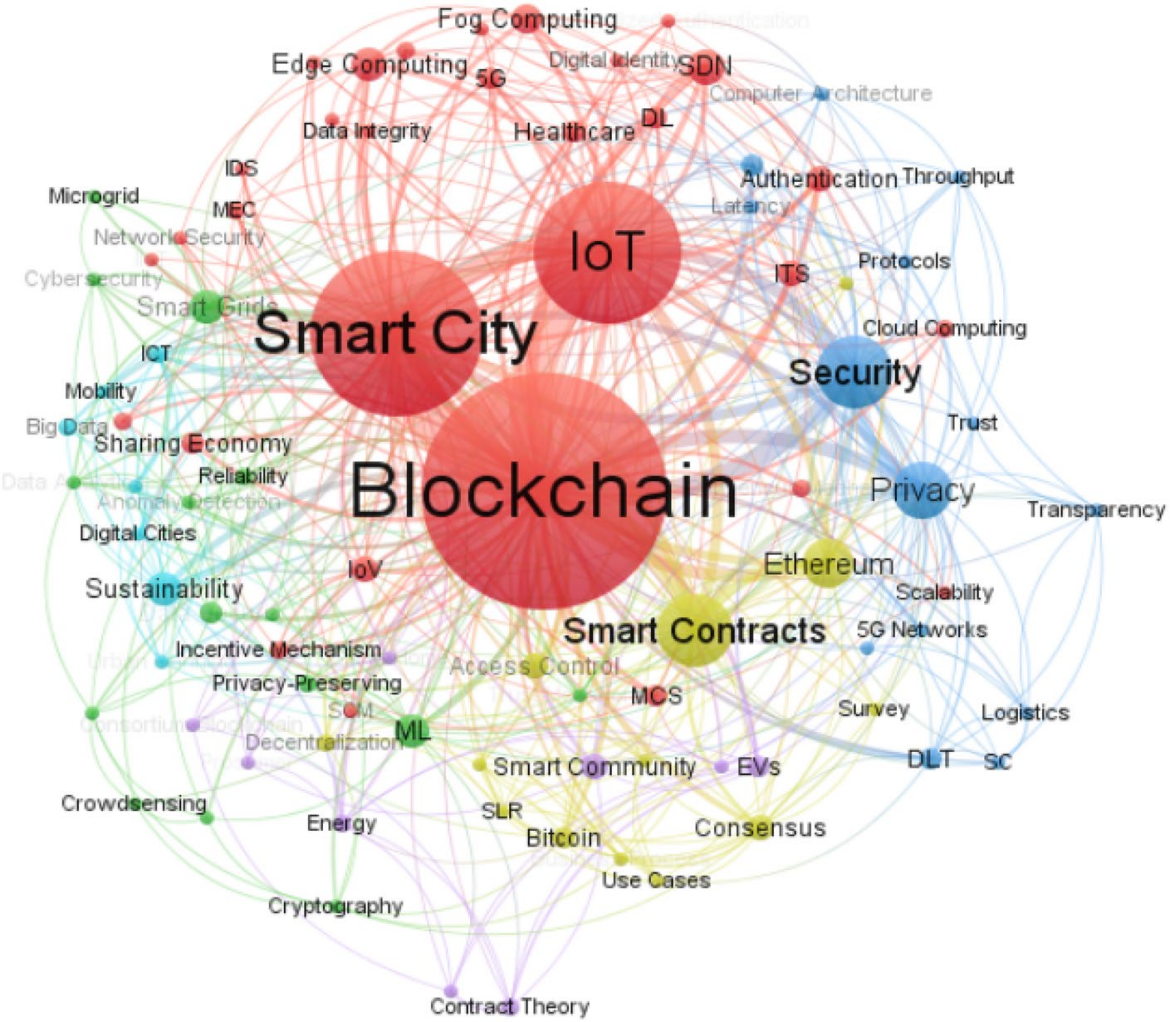
The keyword co-occurrence network

The analysis of keyword co-occurrence network assists researchers in identifying the fundamental topics discussed in a particular research area. In this respect, Zupic and Čater (2015) state that keyword co-occurrence is a useful scientometric technique that enables visualization and displaying the similarities present among frequently co-occurring keywords or topics in the literature. With this bibliometric technique, scholars can obtain a broad idea of the content of a paper and essential information relating to methods, objectives, and viewpoints. To map the keyword co-occurrence network, we pre-treated and adjusted the original keywords whenever it was necessary. For instance, we merged similar keywords such as *"smart city"* and *"smart cities", "blockchain"* and *"blockchain technology"*. After performing some data refinements, we set the threshold of keyword co-occurrence at a minimum of two in VOSviewer and generated the co-occurrence network visualization of content. As illustrated in Fig. 5, six clusters emerged, with 89 nodes appearing in the network. Each node in the visualization constitutes a keyword; the node's size is proportional to the occurrence of the keyword in the reviewed literature. In other words, larger nodes indicate a higher frequency of keyword co-occurrence. Keywords that often co-occur tend to be located near each other in the network. Hence, all authors’ keywords were arranged in six clusters with a different level of significance.

#### The combination of blockchain and IoT in smart cities

Based on the size, the red cluster represents the most significant, containing several keywords that are strongly related to blockchain’s foundational components, the IoT, and a wide range of technologies that could be leveraged in the smart city. These include *"blockchain", “smart city” "IoT", "SDN"* (Software-Defined Networking)*, "Edge Computing", "Fog Computing", "Authentication", "IoV"* (Internet of Vehicles), *"ITS"* (Intelligent Transport System), and *"healthcare"*. The introduction of blockchain technology helps to establish an architecture that ensures improved security and privacy in smart communities (Aggarwal et al. 2019; Bernabe et al. 2019), unmanned aerial vehicle communications (Mehta et al. 2020), IoT devices (Kamran et al. 2020; Singh et al. 2019; Xu et al. 2018), crowdsourcing services (Lin et al. 2020; Stoyanova et al. 2020), healthcare (Dwivedi et al. 2019; Khatoon 2020; Rejeb et al. 2021a, b), and supply chain management (Astarita et al. 2020; Mistry et al. 2020). The high co-occurrence of blockchain with IoT, as shown by the thick edge connecting them in the network, reflects the criticality of these technologies to change the way smart citizens interact, think, live, and manage information. The pervasiveness of IoT technologies can optimize information capture, facilitate data communication, and substantiate the value of blockchain for the development of smart cities. Besides IoT and blockchain, the high occurrence of ITS and healthcare in the author keywords field likely indicates that most of published works focused on the examination of blockchain’s potential for transportation and healthcare activities in the smart city.

Blockchain constitutes a viable solution to overcome several challenges facing transportation activities such as congestion, emissions, and accidents. According to Gupta et al. (2021), blockchain can also be used to mitigate security concerns of connected automated vehicles, including vehicle hacking, traffic congestion, information disclosure, spoofing, eavesdropping, Sybil, replay, data alteration, and DoS attacks. In addition, the technology can underpin a privacy-preserving, open, and trustless architecture for mobility services for citizens (Ahad et al. 2020). By deploying a blockchain-based IoV, it is possible to improve road traffic sustainability by reducing energy consumption, optimizing safety, and minimizing pollution. The introduction of blockchain and the transition from fuel-based vehicles to blockchain-based electric and autonomous vehicles have the potential to advance new business models in which mobility as a service replaces the existing car ownership paradigms. As a result, citizens will be able to benefit from a high level of smart mobility, real-time tracking of public transportation, efficient payment services, sufficient space for parking, and convenient micro-mobility (e.g., finding and tracking scooters or e-bikes with blockchain-based applications).

In the healthcare sector, blockchain is a promising technology that can provide decentralization in healthcare networks, improving data provenance and enhancing network security (Bhushan et al. 2020). Medical professionals can securely store patients’ health data in a blockchain that can be configured to enable interoperability across different healthcare entities. Moreover, blockchain systems can provide real-time access to patients’ medical records and ensure protection against data manipulations that are otherwise difficult to trace, like adding or removing drug allergy information, thereby overcoming critical patient safety and institutional trust concerns (S. Singh et al. 2020a, b). From a patients’ perspective, blockchain can be augmented with identity management tools that provide a secure access framework to medical records and safeguard patient privacy. Data can be accessed only by authorized healthcare actors and entities. To provide appropriate and timely medical interventions and treatments, healthcare organizations could utilize a blockchain-based system to obtain a full record of the patients’ personal health records and diagnoses notes, enabling real-time decision making while reducing patient risk and enhancing the quality of care. Similarly, blockchain can streamline the processes related to medical insurance claims because the technology enables the reduction of the layers of intermediaries involved in the insurance industry (Salha et al. 2019). The ease of secure access to medical data via blockchain can shorten the process of approval of insurance, especially by reducing intermediaries.

From a supply chain perspective, blockchain can be utilized to control and track the flow of medicines and reduce the risk of pharmaceutical counterfeits (Rejeb et al. 2021a, b). Thanks to its fraud-resistant nature, blockchain is capable of reducing pharmaceutical waste and various manipulations of supply chain data. The origin of drugs can be traced efficiently by logging chronological transaction data generated by IoT devices to the blockchain. Therefore, blockchain brings several benefits to the healthcare sector, ranging from timely diagnoses and personalized healthcare services to reduced paperwork and increased pharmaceutical supply chain traceability.

#### The role of machine learning in smart cities

The second significant cluster is the green one, and it comprises terms such as *"ML"* (Machine Learning)*, "Smart Grids", "Data Security", "Privacy-Preserving", "Reliability", "Anomaly Detection", "Crowdsensing"*. According to Tanwar et al. (2020), the combination of blockchain and ML ensures data reliability and security, which is necessary to provide accurate analytics for decision-making processes. Coupled with blockchain, ML can precisely identify patterns, anomalies, and support predictions based on the enormous data generated by IoT applications in the smart city, including transportation, healthcare, energy, and weather forecasting. The integration of ML and IoT has suffered from some limitations, such as the lack of effectiveness in output and the need to embed security from the start (Kaur et al. 2020). For this reason, Salah et al. (2019a; b) believe that blockchain can mitigate these limitations and offer scalability, security, and execution of transactions by consensus protocols (e.g., Proof of Work (PoW), Proof of Stake (PoS)). Kumar et al. (2021) aim to establish trust management, privacy protection, and intrusion detection by fusing blockchain and ML. As such, the authors intend to develop a trustworthy privacy-preserving secured framework for constructing a sustainable smart city. Likewise, Shen et al. (2019) propose a new privacy-preserving training scheme based on blockchain and ML to tackle the issues of data privacy and integrity and to develop a secure training algorithm in multi-part contexts where IoT data is captured from multiple data providers in the smart city. Overall, smart city designers can take advantage of the powerful combination of blockchain and ML to develop secure and privacy-preserving applications.

With the exponential increase in sensors, there is an emphasis on big data and data analytics. These technological advances pave the way for the establishment of data-driven smart cities. Moreover, there is an increased focus on smart grids as a critical feature of emerging energy scenarios with the all-embracing purpose of better balancing energy demand and generation. Smart grids embody a significant improvement in the fundamental structure of power grids, which can be facilitated by blockchain implementation. For instance, blockchain can help address the security and privacy issues encountered during the trading of excess power among smart citizens. In line with Jaiswal et al. (2020), blockchain can be utilized effectively to facilitate electricity trade among user networks. Sadik et al. (2020) posit that blockchain holds the key to the automation of smart grids due to its ability to facilitate energy trading, financing for renewables, energy billing, and metering processes. The technology also incites the joint operation of renewable sources with their energy storage units, the easy assessment of service charge, and the flexible and reliable payments of energy transactions through cryptography. Therefore, blockchain offers more secure energy transactions and trading in a smart city while reducing costs, maximizing utility, and supporting real-time processing of energy trading computation (Chaudhary et al. 2019).

The high frequency of crowdsourcing in the cluster suggests that this paradigm can benefit from the deployment of blockchain in the smart city. To be specific, blockchain has been used to link and automate data processing for mobile crowdsensing, thereby laying the foundation for several smart city applications like smart parking. In this context, Kim and Kim (2020) design a multi-blockchain structure for data management employing mobile crowdsensing technology to develop a smart parking system, which represents a key service for constructing smart cities. Furthermore, by incorporating public–private blockchain, the proposed system is useful owing to the benefits of the public blockchain (e.g., transaction immutability, cryptography, and data integrity) and the private blockchain (e.g., secure data sharing and management). As a result, blockchain can secure information exchange among different participants in mobile crowdsensing networks and achieve decentralization of crowdsensing to prevent the issues that a central platform causes.

#### The technical characteristics of blockchain

The blue cluster is related to the technical characteristics of blockchain technologies such as security, privacy, latency, throughput, transparency, and trust. Interest in the technology stems from the fact that blockchain provides security, confidentiality, and data integrity without the need for a third party to control transactions. Ensuring security is becoming primary for smart city administrators in order to mitigate cybercrimes. To address the security and privacy issues encountered by smart cities, there is a need to integrate blockchain to make sure that certain security loopholes do not continue to affect the rest of the smart city networks. Singh et al. (2020a; b) note that blockchain can be utilized to enhance the security infrastructure of big enterprises as open ledgers can induce fault-proof integration of connected IoT devices in intelligent networks. This prevents problems like data visibility and end-to-end process tracking, transaction automation and checking, real-time data sharing across the network, and IoT network issues. Bhushan et al. (2020) contend that blockchain-based systems require minimum overall security monitoring cost and offer protection from adversaries attempting to gain access to private information or control over the entire network. Moreover, the keyword "AI" appeared frequently in the cluster, illustrating the criticality of this technology to support the development of future smart cities. AI intersects considerably with blockchain to deliver bug-free smart contracts, optimize energy consumption, learn from distributed data sources, improve system resilience, and improve the design of mining hardware to maximize system performance (S. Singh et al. 2020a, b). In turn, blockchain complements AI and ensures data quality and resistance against hacking and human mistakes thanks to its decentralization, immutability, and transparency (Radu 2020). With the emergence of 5G networks, there is a potential to develop vertical applications in the smart city by connecting heterogeneous devices and equipment with substantial enhancements in terms of high service quality, high network capacity, and increased system throughput (Nguyen et al. 2020). 5G networks facilitate the integration of IoT and provide access to the smart city infrastructure through control and virtual network functions (Capossele et al. 2018). The amalgamation of blockchain and 5G allows detecting and preventing various attacks, improving network latency, and providing better connectivity for smart city applications, including smart logistics. In fact, logistics is recognized as the most suitable sector for the introduction of blockchain technology. According to Astarita et al. (2020), the relevance of blockchain for logistics is claimed for its traceability and integration capabilities in supply chain management. It is also argued that enhanced trust and data sharing among supply chain partners are among the virtues of blockchain in logistics. The high frequency of the keyword "Literature Review" indicates that researchers widely used reviews to investigate the security and privacy issues of blockchain and the role of AI and 5G networks in reshaping smart cities. However, a bibliometric approach synthesizing research on blockchain applications in smart cities is still lacking, making this study the first attempt to analyze this topical area using quantitative tools.

#### The role of smart contracts in smart cities

The most frequent keywords in the yellow cluster are "Smart Contracts", "Ethereum", "Access Control", "Consensus", and “Bitcoin”. The collection of these keywords suggests that blockchain supports the programmability and the communication of the multiple heterogeneous, mobile, autonomous and robotics devices used in the smart city through smart contracts. This cluster provides the first and second generations of blockchain applications for developing a ledger that records signed monetary transactions and provides a general-purpose programmable infrastructure using smart contracts (Kosba et al. 2016). Smart contracts represent self-executing computer programs (Szabo 1996) that can be stored within the blockchain and automatically activated if certain conditions are met (Liao and Wang 2018). According to Liao and Wang (2018), blockchain-enabled smart contracts could provide a perfect mixture of security, privacy and ease of use for performing business transactions, exchanging cryptocurrencies (e.g., Ether, bitcoin) and resources. By using smart contracts, blockchain can be leveraged to tap into the underutilized assets of smart cities to generate profits for their inhabitants and strengthen the economy. The technology can accelerate the transition from the smart city economy toward a sharing economy. In their recent study, Rahman et al. (2019) state that blockchain-based sharing economy services can be effectuated by employing smart contracts. The authors further note that with the help of smart contracts, blockchain can automate location-aware agreement logic and provide complex spatio-temporal services (e.g., insurance) to a global level without the need for a central verification body. Moreover, the combination of smart contracts and Ethereum blockchain supports several use cases in the smart city. For example, Salah et al. (2019a; b) apply smart contracts and Ethereum blockchain for food traceability and tracking across agricultural supply chains. In the system, all transactions are kept in the blockchain’s ledger and are connected to a decentralized file system to maintain the targeted level of traceability. In the same vein, Toyoda et al. (2017) leverage Ethereum blockchain and smart contracts to develop a traceability system for the tracking of product information and for identifying forged products. In the context of the automotive industry, Sharma et al. (2019) develop an Ethereum blockchain-based distributed system to ensure on-demand services for customers in smart cities. With the aid of this blockchain, suppliers and manufacturers would be able to protect their products from counterfeits and establish a sustainable automotive ecosystem. Finally, Longo et al. (2019) use an Ethereum blockchain to allow firms to validate the integrity, invariability, and authenticity of data exchanged by other firms, thus precluding companies from sharing inaccurate or unreliable data. Another open-source blockchain that allows the development of smart contracts is Hyperledger Fabric. Using the smart contract of Hyperledger Fabric, Hang and Kim (2020) use this functionality of blockchain to simplify the task management of sensors and verify IoT devices and actuators at runtime. Jamil et al. (2019) present a solution to data integrity management based on Hyperledger Fabric to increase data security in a smart hospital. Sifah et al. (2020) design a system for smart city governance using Hyperledger Fabric as the blockchain platform and operating scheme. As per the study findings, the proposed platform permits the simultaneous tackling of several security issues like trust, privacy, and accountability. It also establishes a healthy smart city environment among government workers. In the supply chain context, Wang et al. (2020) develop a blockchain-based Hyperledger Fabric to solve traceability and fragmentation issues and improve information sharing among business stakeholders. Other use cases of Hyperledger Fabric include education, insurance process improvement, and transportation (Shen and Pena-Mora 2018). Compared to Ethereum blockchains, Hyperledger Fabric-based blockchains are more efficient and able to maintain stable time consumption during smart city transactions (Chiu and Meng 2021).

Beyond Ethereum and Hyperledger Fabric, Bitcoin blockchain also attracts significant attention from smart city researchers. This blockchain is widely used for cryptocurrency and not appropriate for general purpose applications. Nevertheless, being the initial and largest public blockchain, Bitcoin provides a safe and strong payment system that outperforms any other blockchains. In general, Bitcoin blockchain is more suitable for smart city applications that need limited business logic and high security, such as protection of government documents (Beris and Koubarakis 2018), e-voting (Noizat 2015), and machine-to-machine micropayment (Lundqvist et al. 2017).

#### The development of smart community

The purple cluster contains the following keywords; "*Smart Community*", "*EVs*" (Electronic Vehicles), "*Contract Theory*", and "*Energy*". The smart community represents an essential element of the Internet of Energy (IoE), to which blockchain can contribute by securing EV charging, optimizing the allocation of energy resources, and satisfying EV's individual energy consumption preferences (Su et al. 2019). Blockchain has emerged to develop smart communities and optimize their consumption of resources (Alcarria et al. 2018). Aggarwal et al. (2019) posit that blockchain can be used to realize authentication, authorization, security, confidentiality, integrity, non-repudiation, and accountability for real-time smart city applications, which may not be enabled by centralized systems in a smart community context. Blockchain is also a critical element for strengthening transaction security at charging stations of public electric vehicles. Thus, the technology can facilitate electricity trading between charging stations and electric vehicles (Bhushan et al. 2020). In Kang et al. (2017), the authors suggest a consortium blockchain for electricity trading among vehicles and for optimizing prices and the amount of electricity traded. Huang et al. (2018) develop a trading model based on blockchain to store the transaction information between electric vehicles and charging stations and to automate trading activities. Therefore, blockchain contributes to the development of green transportation systems by streamlining transparent and decentralized trading processes between vehicles and charging stations. Demand information of electric vehicles (e.g., necessary energy amounts, geographic region, time interval) and charging stations (e.g., location, pricing) can be maintained by blockchain; therefore, electric vehicles can make more effective decisions, particularly concerning the selection of the optimal charging station. Furthermore, blockchain encourages P2P transactions and ushers in new prosumer markets, where transactive energy systems are the predominant paradigm. In such systems, utilities and smart homes in the city move from passively consuming energy and receiving demand-response calls to actively trading energy and ancillary services on blockchain and even to developing locally efficient market clearing prices (Qi and Shen 2019). The use of blockchain-based P2P energy transaction is able to establish sustainable energy ecosystem between prosumers, customers, and existing energy suppliers (Shojaei et al. 2019). Finally, the keyword "Smart Home" is indicative of blockchain’s potential to monitor its resources, ensure security and privacy, and increase home automation (Li et al. 2019).

#### Blockchain as a driver for sustainable smart cities

Finally, the last cluster consists of the following keywords: “*Sustainability*”, “*Big Data”, “ICT”, “Citizens”*. Terms shown in this cluster reveal the role of blockchain to incorporate sustainability in the infrastructure of smart cities. In general, a smart city is orchestrated to enable operational efficiencies, increase environmental sustainability efforts, and improve citizens’ quality of life. By utilizing blockchain in smart mobility, there is a chance for less vehicles and road traffic, helping to reduce air pollution and fossil fuel emissions (Wong et al. 2020). To further strengthen the sustainability of urban mobility, Jaffe et al. (2017) propose an incentive model that could be used by blockchain to prompt citizens in cycling and to recompense commuting practices. The reward system also involves citizens choosing public transport or shared rides in the smart city. Due to the increasing urbanization, it is crucial to devise effective waste management policies to enforce the sustainable dimension of smart cities. Related to waste, the coupling of blockchain, IoT sensors and AI has the potential to assist in waste management processes, including waste collection, disposal, and recycling. Through the implementation of blockchain, civil engineers can benefit from reliable big data to improve urban resources and services (Berglund et al. 2020). In the bargain, smart cities can take advantage of blockchain to advance economic, environmental, and social sustainability. In response, this can support a country’s commitment to meet sustainable development goals. While the literature emphasizes that blockchain is ready to assimilate a key role in the sustainable development of smart cities and enhance people’s life conditions (Shen and Pena-Mora 2018), sustainability still represents the furthest research topic from the mainstream of the analyses represented by other clusters. Consequently, there is a need for additional studies ascertaining the sustainability outcomes of blockchain in the smart city (Table 1).

**Table 1:** Top 10 most frequent keywords in each cluster

Rank	Cluster 1	Cluster 2	Cluster 3	Cluster 4	Cluster 5	Cluster 6
1	Blockchain	ML	Security	Smart Contracts	Smart Community	Sustainability
2	Smart City	Smart Grids	Privacy	Ethereum	EVs	Big Data
3	IoT	Data Security	AI	Access Control	Contract Theory	ICT
4	SDN	Privacy-Preserving	DLT	Consensus	Energy	Citizens
5	Edge Computing	Reliability	5G Networks	Bitcoin	Consortium Blockchain	Digital Cities
6	Fog Computing	Anomaly Detection	Computer Architecture	Decentralization	Demand Response	Mobility
7	Authentication	Crowdsensing	Latency	Authorization	Prosumer	Urban Planning
8	IoV	Cryptography	Literature Review	Business Process	Reputation	
9	ITS	Cybersecurity	Logistics	Hyperledger Fabric	Smart Home	
10	Healthcare	Data Analytics	Protocols	P2P Computing		

IoT: Internet of Things/SDN: Software-Defined Network/IoV: Internet of Vehicle/ITS: Information Transportation System/ML: Machine Learning/AI: Artificial Intelligence/DLT: Distributed Ledger Technology/P2P Computing: Peer-to-Peer Computing/EV: Electrical Vehicles/ICT: Information and Communication Technologies

### Article co-citation network

**Fig. 6:**
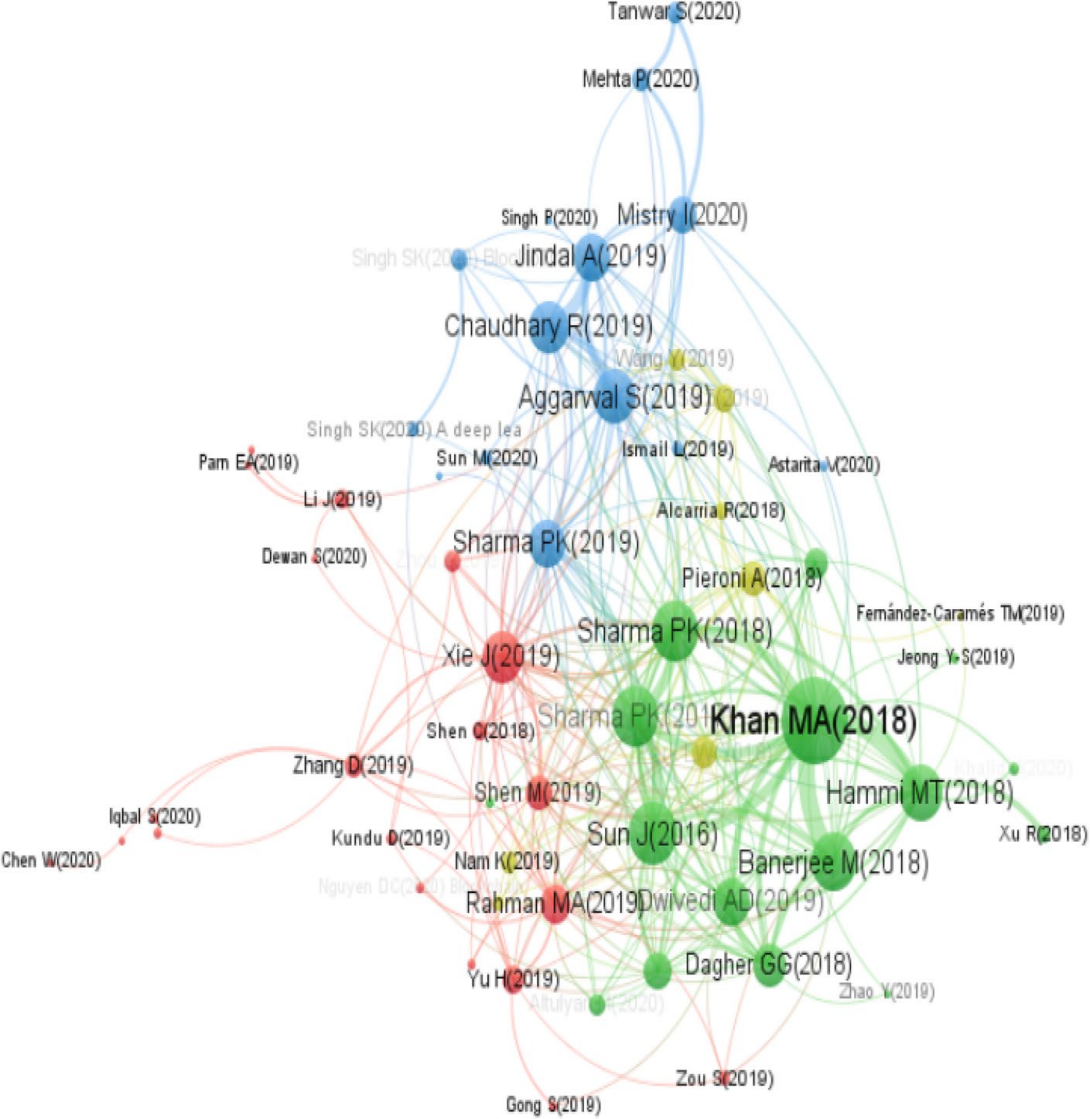
Article co-citation network

An article co-citation network represents an essential type of co-citation analysis. The origin of this method can be traced to the work of Small (1973), who recommends the analysis of the network of co-cited references. In this method, the units of the network analysis are the articles, and co-citation clusters constitute the underlying intellectual structures of a particular field. Consistent with Chen et al. (2010), article co-citation enables researchers to interpret the nature of the cited articles in the cluster and the interlinks between the clusters. The purpose of this study was to conduct an article co-citation analysis to determine the structure of the most relevant contributions to the research area of blockchain and the smart city. Figure 6 presents the findings of the article co-citation network extracted from VOSviewer. As shown, the highly co-cited pair of articles are those connected with thick arcs. A pair of co-cited articles occur when two articles are cited together in a single article. The thick arcs indicate a strong relationship between these articles and suggest similarities regarding specific topics within the field of blockchain and smart city (Batistič et al. 2017; Small 1973; Zupic and Čater 2015).

On the other hand, thin arcs signify a weak co-citation association between co-cited articles and the lack of content similarities (Mishra et al. 2017, 2018). For example, the thick arc connecting Khan and Salah  (2018) and Hammi et al (2018) suggests that these articles have a strong co-citation relationship and share common ideas and/or related concepts. A similar pattern can be observed between Sharma et al. (2017) and Sharma and Park (2018). In contrast, the thin arc between Hammi et al. (2018) and Mistry (2020) reflects a weak co-citation strength, and thus the lack of content similarities (Mishra et al. 2017, 2018).

Setting a threshold of at least two citations per article, only 58 nodes of the most co-cited articles are displayed in the co-citation network in Fig. 6. The radius of a node gives an idea about its total link strength which is the sum of link strengths of this node over all the other nodes. Moreover, four clusters were generated by VOSviewer. In terms of the total number of nodes, the red cluster is the most significant one. This cluster provides a general overview of blockchain applications in the smart city (Rahman et al., 2019; E. Xie et al., 2006) and the role of the technology in optimizing the efficiency and strengthening the security and privacy of various smart city applications such as IoT (Gong et al. 2019; Shen et al. 2019; Viriyasitavat et al. 2019), IoV (Chen et al. 2020; Iqbal et al. 2020; Yin et al. 2020; Zhou et al. 2019), and big data (Yu et al. 2019). As a result, researchers interested in understanding the potentialities and challenges of blockchain for the smart city in general, and the combination of the technology with IoT and IoV in particular, may refer to the articles grouped in the red cluster. The green cluster groups all authors belonging to the same area of research and studying the potentials of blockchain for securing the security and privacy of IoT transactions and information sharing (Banerjee et al. 2018; Khan and Salah 2018; Makhdoom et al. 2020; Xu et al. 2018). Furthermore, the blue cluster revolves around the potentials of blockchain solutions for smart communities (Aggarwal et al. 2019), energy trading (Chaudhary et al. 2019; Jindal et al. 2019), transportation (Astarita et al. 2020; Mehta et al. 2020; Sharma and Park 2018), artificial intelligence (Singh et al. 2019, 2020a, b), and construction (Sun and Zhang 2020). Lastly, the yellow cluster primarily comprises all significant works associated with the application of blockchain for smart energy (Alcarria et al. 2018; Park et al. 2018; Pieroni et al. 2018; Su et al. 2019; Wang et al. 2019), smart tourism and entertainment (Liao and Wang 2018; Nam et al. 2019), and smart education (Fernandez-Carames and Fraga-Lamas 2019). This cluster mostly groups all authors discussing the ability of blockchain to secure energy transactions and create a transparent system for energy supply and trading.

The privacy-by-design nature of blockchain is well-suited for smart city applications such as healthcare (Dagher et al. 2018; Dwivedi et al. 2019), data sharing (Makhdoom et al. 2020), and sharing economy services (Rahman et al. 2019). Regardless of healthcare policies, blockchain could be implemented in the smart city to authenticate and secure the sharing of medical records among the healthcare sector stakeholders. The storage of sensitive medical data in blockchain can be helpful in situations of emergency, crisis, and pandemics, such as the recent COVID-19 outbreak. However, these application fields have received less attention, especially from the empirical point of view.

From Fig. 6, it appears that the most pertinent and highly cited study is that by Khan and Salah (2018), who conducted a review on IoT security, blockchain solutions, and the challenges faced by IoT technologies. Three articles with more than 170 citations stand out for their number of citations (See Table 2). The study of Khan and Salah (2018) has received the highest number of citations and gained a significant level of academic attention. The authors of this study performed a comprehensive review of IoT and blockchain technologies. Taken together, these technologies are expected to create several opportunities for smart cities and introduce new challenges. The second study by Sharma et al. (2017) received 189 citations. The authors proposed a vehicle network architecture based on blockchain in the smart city. Scholars recognize the importance of the proposed system and the ability of blockchain to optimize the use of infrastructure resources and create intelligent transportation systems. A study by Sun et al. (2016) received 172 citations. This study developed a conceptual framework and analyzed the benefits of blockchain for supporting communal sharing and renovating services in the smart city. Taking a closer look at all these articles, it is evident that blockchain holds the potential to add value in different smart city sectors and applications, including integration with smart IoT technologies, smart transportation, smart services, smart healthcare, smart grids, and smart energy. Overall, since most influential articles are recent, it may take a few years before the asymptotic behaviour of their citations will become known.

**Table 2:** Annual number of growth of citations received for the top ten highly cited articles

Authors	Year	Title	2016	2017	2018	2019	2020	Total
Khan and Salah (2018)	2018	IoT Security: Review, Blockchain Solutions, and Open Challenges	0	0	35	240	273	548
Sharma et al. (2017)	2017	Block-VN: A Distributed Blockchain Based Vehicular Network Architecture in Smart City	0	3	31	71	84	189
Sun et al. (2016)	2016	Blockchain-Based Sharing Services: What Blockchain Technology can Contribute to Smart Cities	1	4	24	66	77	172
Dagher et al. (2018)	2018	Ancile: Privacy-Preserving Framework for Access Control And Interoperability of Electronic Health Records Using Blockchain Technology	0	0	10	52	82	144
Hammi et al. (2018)	2018	Bubbles of Trust: A Decentralized Blockchain-Based Authentication System for IoT	0	0	4	53	79	136
Banerjee et al. (2018)	2018	A Blockchain Future for Internet of Things Security: A Position Paper	0	0	10	54	65	129
Dwivedi et al., (2019)	2019	A Decentralized Privacy-Preserving Healthcare Blockchain for IoT	0	0	0	43	81	124
Sharma and Park (2018)	2018	Blockchain Based Hybrid Network Architecture for the Smart City	0	0	3	35	54	92
Xie et al. (2019)	2019	A Survey of Blockchain Technology Applied to Smart Cities: Research Issues and Challenges	0	0	0	8	64	72
Su et al. (2019)	2019	A Secure Charging Scheme for Electric Vehicles with Smart Communities in Energy Blockchain	0	0	3	20	45	68

## Discussion and research implications

### Discussion

Our findings reveal that the concept of blockchain has rapidly evolved over recent years. Due to the tremendous advances in technology, a wide range of applications have emerged, including those that significantly impact the way smart cities are designed and managed. The use cases of blockchain in the smart city have become a priority topic for investigation by scholars, practitioners, and governments worldwide. Several attempts were made to showcase the promising capabilities of blockchain technology for smart city applications. To date, the proliferation of big data and the rapid evolution of IoT technologies have helped to accelerate the development of smart cities. While these innovations have played a critical role in reshaping different areas of human life, influencing many sectors like healthcare, transportation, education, energy, and services, they generate an array of challenges such as security threats (Ismail and Materwala 2019; Khan and Salah 2018), privacy issues (Bernabe et al. 2019), and technical inefficiencies (Rathore et al. 2019).

#### Blockchain for secure smart city applications

The complexity underlying the administration and management of a smart city through its infrastructure and systems creates a substantial amount of sensitive data that necessitate a secure and large storage environment. To mitigate cyber threats and ensure safe data capture, data storage and data access in smart cities, blockchain’s distributed ledger, encryption, and immutability features can provide a foundation layer for various citizen and business interaction applications with increased efficiency, improved security, and enhanced privacy. Features of blockchain such as enhanced reliability, unforgeability, and fault tolerance, make the technology an effective tool for authentication problems (Khalid et al. 2020) and content integrity protection (Xie et al. 2019). Therefore, the vision of the smart city can be achieved with the use of blockchain (in combination with other technologies) as the technology compliments a city’s digital transformation with enhanced security and privacy, both significant pillars of smart cities (Ahad et al. 2020).

Furthermore, the integration of blockchain in smart cities can guarantee secure and real-time data transmission between smart city technologies (e.g., IoT, cyber-physical systems, drones, robotics) without the need for a central entity that manages and controls all the transmitted data. The security feature of blockchain helps facilitate data transfer (Dwivedi et al. 2019; Mehta et al. 2020), but also enables seamless interactions between users of autonomous systems in a smart city environment. For instance, the combination of various devices applied in various smart city applications (e.g., smart transportation, smart healthcare, smart factory, smart energy) can benefit from the peer-to-peer network for authentication and resilience against attacks. By taking advantage of these merits, Singh et al. (2019) argue that blockchain can support the connection of artificial intelligence (AI)-enabled data centres and allow decentralized and secure big data analysis. Therefore, researchers of smart city applications that are based on data generated and collected by various technological components have recognized the critical hardware and software security threats that have arisen with the deployment of new technologies. As per this review’s findings, the nature of embedded security and trust stresses the importance of blockchain to enhance the integrity of smart city systems to overcome perceived security risks. As a result, the use of blockchain to better manage the risks related to system security may increase citizens’ acceptance and usage of information technology-enabled services and maximize the effectiveness of the smart city initiative.

#### Blockchain for private smart city transactions

Besides robust security, blockchain technology provides privacy measures for the massive amounts of data generated in the smart city, including weather, energy, healthcare, manufacturing, and services data. New technologies can pose significant privacy concerns without reasonable privacy measures due to the massive aggregation of location-based information and their transfer over smart city networks. However, with the support of blockchain, it is possible to tackle the complexity and privacy issues of using distributed databases (Ismail and Materwala 2019). Blockchain provides a practical scheme, which preserves data privacy and ensures authorized access to different data types, including health, smart cars, smart energy, and financial details (Makhdoom et al. 2020). Privacy applications of blockchain can prevent breaches, protect personal data (Astarita et al. 2020), and embed privacy in products and services (Oliveira et al. 2020). According to several studies, blockchain acts as an innovative and effective safeguard to the privacy of transactions due to its authentication and encryption mechanisms (Altulyan et al. 2020; Chaudhary et al. 2019; Khan et al. 2020; Nam et al. 2019; Treiblmaier et al. 2020). With fewer worries about their privacy, citizens can rely on blockchain-enabled applications to protect their personal information.

#### Blockchain for smart city sustainability

The integration of blockchain technology can transform every sector of the smart city. Such transformation can bring several smart city improvements in more resilient information systems, proper governance models, sustainable use of resources, intelligent management and allocation of natural resources and city facilities, and enhanced quality of life. The improvement of sustainability has always been one of the desired targets in the smart city. By integrating sustainability with blockchain technology, the smart city can drive sustainable development and growth from an economic, social, and environmental perspective. For example, the challenges associated with energy consumption can be addressed by blockchain. The technology can be used as a foundation to develop a peer-to-peer (P2P) energy-transaction platform to connect energy resources and home appliances and provide low-cost energy at all times and locations (Park et al. 2018). The inclusion of sustainability aspects in a smart city also involves adopting blockchain for smart waste management, the reduction of environmental impacts (Berglund et al. 2020), and the preservation of ecosystem resiliency (Hammi et al. 2018). Blockchain is expected to support the smart city paradigm, foster green strategies, and enhance urban sustainability. Smart cities can benefit from the emergence of blockchain to increase efficiencies and reduce the strain on scarce resources. Consequently, blockchain implementation enables system designers to create a sustainable smart city, which is the engine of economic efficiency, growth, and social inclusiveness (Berglund et al. 2020). This study shows that blockchain can hasten moves towards a more sustainable smart city, in which the wise management of resources is aligned with social inclusion and urban liveability.

#### The fusion of blockchain and IoT in the smart city

The combination of blockchain and IoT provides more robust and secure platforms for smart sensors and devices to interact seamlessly within the smart city environment. Also, blockchain technology contributes to developing ‘security-by-design’ and intelligent IoT systems that can be used in smart grids, smart homes, smart transportation, smart energy, and smart healthcare. The phenomenal proliferation of IoT has enabled ubiquitous connectivity and intelligent automation, contributing to the development of smart cities. To advance IoT-enabled smart cities, blockchain can play a critical role in improving IoT networks, achieving fault tolerance, optimizing resource consumption, and enhancing data processing performance (Liu et al. 2020). The combination of blockchain and IoT is an enabler for transparency (Mistry et al. 2020), seamless data integration (Viriyasitavat et al. 2019), and increased security (Rathore et al. 2018). Empowered by blockchain technology, IoT can enable trust-free transactions, automation, and secure machine-to-machine communications between IoT devices. This paper, therefore, argues that in a smart city, blockchain can respond to several needs for the secure interconnection between devices and citizens, efficient sharing of information, resources, and services. The application of blockchain technology for the smart city can create reliable storage of big data, resulting in better smart city services. Using blockchain data, decision-makers would have the opportunity to effectively plan for enhancing smart city services, expanding resources, and making informed decisions.

#### The challenges of blockchain in the smart city

In advancing the discussion further, a relevant follow-up question we asked is "What are the challenges of adopting blockchain in the smart city?" Despite the enablers of blockchain for smart cities, as evidenced in the reviewed literature, the relative immaturity and lack of wide-scale implementations of the technology constitute one of the significant systems-related barriers (Astarita et al. 2020). This technological immaturity may increase the risk and uncertainty level of citizens, smart city designers, and decision-makers, thereby reducing or delaying the adoption of blockchain. However, the technology's infancy does not constitute a problematic issue since blockchain applications are expected to mature with time.

Another pressing challenge of blockchain deployments in the smart city is the limited scalability of blockchain systems (Liu et al. 2020). For instance, the early generations of blockchain implementations are notoriously characterized by low scalability (Scekic et al. 2019). Ismail and Materwala (2019) argue that the low scalability of blockchain is essentially caused by data redundancies and increasing computational and communication overheads. From the perspective of storage, throughput, and networking, the existing blockchain designs still suffer from scalability issues. Thus, more decentralized, energy-efficient, high transaction throughput, and highly scalable blockchain consensus algorithms are required. The realization of these needs and the development of scalable blockchains will help overcome the misalignment between the existing protocols and the customer services and meet the requirements of large-scale collaborative ecosystems in the smart city (Ismail and Materwala 2019). Therefore, studies proposing practical solutions for blockchain scalability in the smart city are welcome, considering security, reliability, and fine-grained decentralized access control (Mistry et al. 2020).

Another challenge of blockchain implementations in the smart city refers to the lack of standards and acceptance at the official level. So far, there are no technology standards that help blockchain integration with other technologies such as the IoT and 5G networks (Mehta et al. 2020; Mistry et al. 2020). Ahmed et al. (2020) note that current IoT frameworks rely on unified or expedited standards with enormous computational and capacity limits. Given that the communication between devices and nodes with multiple software interfaces necessitates a shared platform and protocol to facilitate data transfer, it is urgent to establish standards and protocols globally in the smart city (Tiwari and Batra 2021). As a result, the adoption of blockchain in smart cities requires proper governance, policies, rules, and guidelines for the effective deployment of the technology. Without standards and regulations, the smart city’s systems would not be able to interoperate, making it impossible to share information across platforms. Likewise, the development of efficient and collaborative smart city applications will become a complicated task, and undesirable consequences (e.g., data inconsistencies and fragmentation, software management issues, operational inefficiencies) may arise when dealing with configured devices (Mistry et al. 2020).

### Research implications

#### Theoretical implications, limitations and future research directions

In this study, we conducted a systematic bibliometric review to generate some relevant insights on blockchain research in the smart city context. Nonetheless, our bibliometric analyses have several limitations:

- The review results are based on a short period of the publications involved (2016–2020) and are restricted to specific subject areas. Although the list is comprehensive and covers leading academic journals with highly cited and co-cited articles, the collection of studies is not exhaustive. Several research studies are expected to be conducted in the foreseeable future as blockchain technology has gained worldwide traction.
- We only conducted one method of co-citation analysis, namely, article co-citation analysis. Many different methods exist to perform co-citation analyses, such as bibliographic coupling, author co-citation analysis, and co-authorship analysis.
- The results of the review were determined by the keywords used for the search and collection of studies. Although we are confident that we surveyed an extensive knowledge base, there might be articles that are potentially relevant to the scope of the study but not captured.
- We have only selected journal articles, ignoring other vital sources of knowledge such as conference proceedings, books, and book chapters. Though the selected articles provide valuable insights for academics and practitioners, non-journal articles may offer interesting insights that are not covered in our reviewed literature.
- An important step in conducting a bibliometric study is the preparation of data. Even though numerous databases and electronically accessible sources can be used in bibliometric studies, most technology-oriented analyses choose only one database as a source (Suominen and Seppänen 2014) because the bibliometric software (i.e., VOSviewer) is only able to read data from one database and cannot merge data from multiple databases (Yeung et al. 2019). Therefore, future researchers are encouraged to use the Web of Science database in their bibliometric studies, which may imply different clustering results and novel insights.

This study applied bibliometric techniques, including keyword co-occurrence network analysis and article co-citation analysis, to investigate the core content of blockchain research in the context of smart cities and identify the intellectual structure of this nascent literature. The overall analysis comprised the evaluation of 148 articles published from 2016 to 2020. This reveals that blockchain technology has attracted increasing attention from scholars who are active in the smart city research domain. Blockchain technology is expected to penetrate the foundations layers of smart cities and pave the way for more innovative, secure, and smarter applications. The use of blockchain is not without its challenges, given the embryonic nature of the technology and its technical limitations. However, we believe that blockchain in the smart city presents a unique opportunity for researchers to examine the applicability of the technology in different smart city sectors and propose workable solutions that contribute to the wide-scale implementation of the technology. This review might inspire researchers to study further the integration of blockchain, big data, and the IoT.

Moreover, from a theoretical perspective, several theoretical lenses can be applied to smart cities. Social exchange theory (SET) is one of the oldest theories on social behaviour and interactions of individual and the exchange of tangible and intangible resources. It may inform researchers on the evolution of human behaviours and their technology-enabled interactions in smart cities. Further, researchers could apply a transaction cost economics (TCE) lens to analyse the potential efficiencies created by using blockchain and other technologies to enable smart cities.

Finally, we argue that blockchain has the potential to offer fruitful avenues in the field of smart city and urban research. We submit that the characteristics of the technology are encouraging and open windows for future research. The researchers may be interested in exploring the following knowledge gaps:

- Blockchain technology can redefine the economic, social, and environmental dimensions of sustainability in the smart city environment.
- Blockchain technology combined with IoT and big data supports the vision for smart cities.
- Blockchain technology can help mitigate the problems caused by urban population growth by improving the competitive profile of the city.
- Blockchain technology can be a valuable solution to promote participatory governance, fuel sustainable economic growth, and enable effective management of natural resources.
- Blockchain technology can create effective sharing economy models in the smart city and optimize efficiencies in meeting the demands for city services.
- Blockchains can be used for simultaneous assurance of confidentiality, integrity/non-repudiation, and accessibility of content.
- Blockchain technology can drive significant operational efficiencies and free up financial resources in smart cities.
- Blockchain technology can automate and streamline public sector procurement and reduce the risk of fraud in supply contracts.

#### Managerial implications

We underline the importance for smart city designers and practitioners operating in the urban sector to be updated with blockchain technology and the different solutions and applications that could be employed to achieve sustainable smart cities. Smart city designers are recommended to focus on technical features of blockchain while responding to citizens’ concerns in terms of privacy, security, and access control. Capitalizing on blockchain technology can accelerate the smart city movement, ensure the proper functioning of cities, and meet the current and emerging needs of citizens. Nations that are striving to make their cities smarter might find blockchain capable of unleashing the innovative and competitive potential of the city.

Moreover, we stress the importance of best practices and critical success factors in deploying effective policies and strategies to spread blockchain initiatives in smart cities. It is also imperative that programmers, network developers, and smart city designers engage in an ongoing discussion to understand blockchain's finer points and anticipate and remove loopholes that would unavoidably emerge. For governments challenged by the complexities of transforming smart city initiatives into practice, they can apply blockchain for customized smart city solutions, including transportation, energy management, and city surveillance, to satisfy local needs and meet development agendas. Hence, policymakers are advised to rethink the role of technology to foster the economic, environmental, and social strategies and substructures for the practical realization of sustainable smart cities.

## Conclusions

The rapid growth of global population along with an increasing urbanization process have placed pressure on the economic, environmental, and social sustainability of cities. As a result, the smart city paradigm is suggested to leverage modern technologies in a smart fashion so as to construct a livable urban environment and enhance the quality of citizens’ lives. Nevertheless, smart cities are still endangered by a wide array of security and privacy threats. With the emergence of the blockchain, these proliferating issues can be effectively overcome thanks to the intrinsic properties of the technology, including immutability, transparency, auditability, and decentralization. By integrating blockchains, cities are expected to become able to realize sustainable targets because the technology can support several smart city sectors such as healthcare, transportation, logistics and supply chain management, and public administration. While weak citizen involvement, poor governance, and threats of system failure are the main concerns hampering the development of sustainable smart cities (Aghimien et al. 2020), digitalization of administration, citizenship, smart city activities through blockchain can bring several advantages in terms of system transparency, security, resilience. Moreover, the alignment of blockchain with sustainability goals can help cities encounter the degradation of infrastructure and resources by supporting the development of smart applications that alleviate traffic congestion, reduce global warming, and cater to the needs of the citizens.

Drawing on bibliometric tools and techniques, we conducted an extensive review of literature on blockchain and the smart city over 2016 and 2020. We generated interesting insights into the evolutionary patterns of blockchain-smart city research, the major contributing countries, and the scientific journals taking part in advancing this emerging field. Through the keyword co-occurrence and article co-citation networks, we identified the core content of the retrieved literature and the influential studies contributing to the conceptual development of the field. For instance, the fusion of blockchain and IoT is shown to boost security in accessing the data collected by sensors and preserving data integrity for IoT-enabled smart city systems. Blockchain also provides privacy for the implementation of machine learning algorithms in the smart city. To gain significant insights from IoT devices, machine learning can be utilized during data analysis, and its outcomes can be maintained in blockchain for secure storage and sharing. Benefitting from the technical characteristics of blockchain, smart contracts can be applied in numerous trading applications due to their automation, which offers transparent and reliable data sharing among service providers and network participants. Finally, blockchain is found to empower smart communities and foster sustainability in smart cities.

To our best knowledge, this is the first study attempting to conduct a bibliometric review on blockchain and the smart city, unravel the content of this research field, and classify co-cited works. Even though these tools have been applied in other fields, there is a lack of knowledge regarding the investigation of blockchain applications in the smart city using bibliometrics. Therefore, we believe that the current research has been unique, offering significant contributions to theory by proposing fertile research areas. We did not cover all the potential applications of blockchain, as we were concerned only with their impact on the smart city sectors. We hope the trajectory of the research described herein provides food for thought and encouragement for scholars to investigate further the intricacies of adopting blockchain in smart cities and urban developments.
